# A novel PHD-finger protein 14/KIF4A complex overexpressed in lung cancer is involved in cell mitosis regulation and tumorigenesis

**DOI:** 10.18632/oncotarget.14962

**Published:** 2017-02-01

**Authors:** Lin Zhang, Qin Huang, Jiatao Lou, Liangjian Zou, Yiguo Wang, Peng Zhang, Guang Yang, Junyi Zhang, Lan Yu, Dai Yan, Chenyi Zhang, Jing Qiao, Shuting Wang, Sai Wang, Yongdong Xu, Hongbin Ji, Zhengjun Chen, Zhe Zhang

**Affiliations:** ^1^ Key Laboratory of Systems Biology, Shanghai Institute of Biochemistry and Cell Biology (SIBCB), Shanghai Institutes for Biological Sciences (SIBS), Chinese Academy of Science (CAS), Shanghai, China; ^2^ Shanghai Lung Tumor Clinical Medical Center, Chest Hospital Affiliated to Shanghai Jiao Tong University, Shanghai, China; ^3^ Institute of Cardiothoracic Surgery, Changhai Hospital Affiliated to The Second Military Medical University, Shanghai, China; ^4^ Tsinghua-Peking Center for Life Sciences, School of Life Sciences, Tsinghua University, Beijing, China; ^5^ Shanghai Pudong Hospital, Fudan University Pudong Medical Center, Shanghai, China; ^6^ Cancer Research Center, Shanghai Xu-Hui Central Hospital, Shanghai Clinical Center, CAS, Shanghai, China; ^7^ School of Life Science and Technology, Shanghai Tech University, Shanghai, China; ^8^ CAS Center for Excellence in Molecular Cell Science, SIBCB, SIBS, CAS, Shanghai, China; ^*^ These authors have contributed equally to this work

**Keywords:** PHF14, KIF4A, lung carcinogenesis, mitosis, biomarker

## Abstract

The plant homeodomain (PHD) finger-containing proteins have been implicated in many human diseases including cancer. In this study, we found that PHF14, a newly identified PHD finger protein, is highly expressed in lung cancer. The high expression level of PHF14 was associated with adenocarcinoma and poor survival in lung cancer patients. Knocking down PHF14 suppressed cancer cell growth and carcinogenesis, while over-expressing PHF14 promoted cell proliferation. During cell division, PHF14 directly bound to and co-localized with KIF4A (a nuclear motor protein involved in lung carcinogenesis) to form a functional complex. Similarly to the effect of KIF4A depletion, silencing PHF14 in several cell lines caused cell mitotic defects, prolonged M phase, and inhibited cell proliferation. What's more, these two proteins had a synergistic effect on cell proliferation and were significantly co-overexpressed in lung cancer tissues. Our data provide new insights into the biological significance of PHD finger proteins and imply that PHF14 may be a potential biomarker for lung cancer.

## INTRODUCTION

The plant homeodomain (PHD) finger is present in a variety of eukaryotic proteins that are involved in chromatin-mediated transcriptional regulation and chromatin dynamics [1–3]. Abnormal expression or mutations in genes encoding PHD finger-containing factors are known to be involved in the pathogenesis of various human diseases including cancers [4]. For instance, recurrent gene fusion occurs between the *PHF1* and *MEAF6* gene in endometrial stromal sarcoma [5]. Ubiquitin-like with PHD and ring finger domains 1 (UHRF1) has been reported to be overexpressed in various cancers such as breast cancer [6] and lung cancer [7]. *ING* (Inhibitor of Growth) genes, which function as tumor suppressors by maintaining genome stability, regulating DNA repair, and restricting cell proliferation, have been found to be downregulated, lost or misregulated in multiple malignancies [8, 9]. In addition to recognizing histone tails, PHD fingers have been implicated in binding to non-histone proteins in several reports, thus expanding their roles as transcriptional regulators and signaling components [10]. Pygopus (Pygo) is a good example. Its binding to BCL9 is essential for Wnt responses during development [11].

PHF14 (PHD finger protein 14) belongs to the PHD finger protein family. As a newly identified protein, its function is far from clear. PHF14 is a chromatin-binding protein, containing four putative PHD fingers and two coiled-coil regions, and interacts with histones via its PHD1 and PHD3 domains [12], which indicates its potential role in epigenetic regulation. Depletion of *PHF14* in mice results in neonatal lethality due to respiratory failure and poor-developed alveoli [12, 13]. PHF14 might be a negative regulator for platelet-drived growth factor receptorα (PDGFRα) expression in mouse mesenchymal cells in PHF14−/− lung tissue [13]. *PHF14*-knockout mice also exhibited severe disorders of tissue and cell structures in multiple organs including lungs and kidneys. Several other studies have implicated PHF14 in diseases including cancers. Overexpression or haploinsufficiency of *PHF14* has been detected in patients with Dandy-Walker malformation [14]. In a colon cancer cell line HCT-116, a bi-allelic inactivating mutation of *PHF14* has been identified [15]. Recently, the homozygous deletion of *PHF14* has also been identified in a human biliary tract cancer cell line (OZ) [16]. PHF14 may have multiple functions in gene regulation, cell proliferation, and tumor development.

In the present study, we found that PHF14 was highly expressed in lung cancer. Its high expression level was associated with poor survival of lung cancer patients. Depletion of PHF14 inhibited lung cancer cell colony formation in soft agar and tumor formation in nude mice. By employing proteomic approaches, we identified kinesin family member 4A (KIF4A), which was overexpressed in lung cancer [17], as a potential PHF14 binding-protein. Our data further demonstrated that PHF14 forms a physiological complex with KIF4A and regulates mitosis and cell proliferation. Both two genes were significantly overexpressed in lung cancer tissues /lung cancer cell lines and were involved in lung tumorigenesis.

## RESULTS

### PHF14 overexpression is associated with poor prognosis of NSCLC

**Figure 1:**
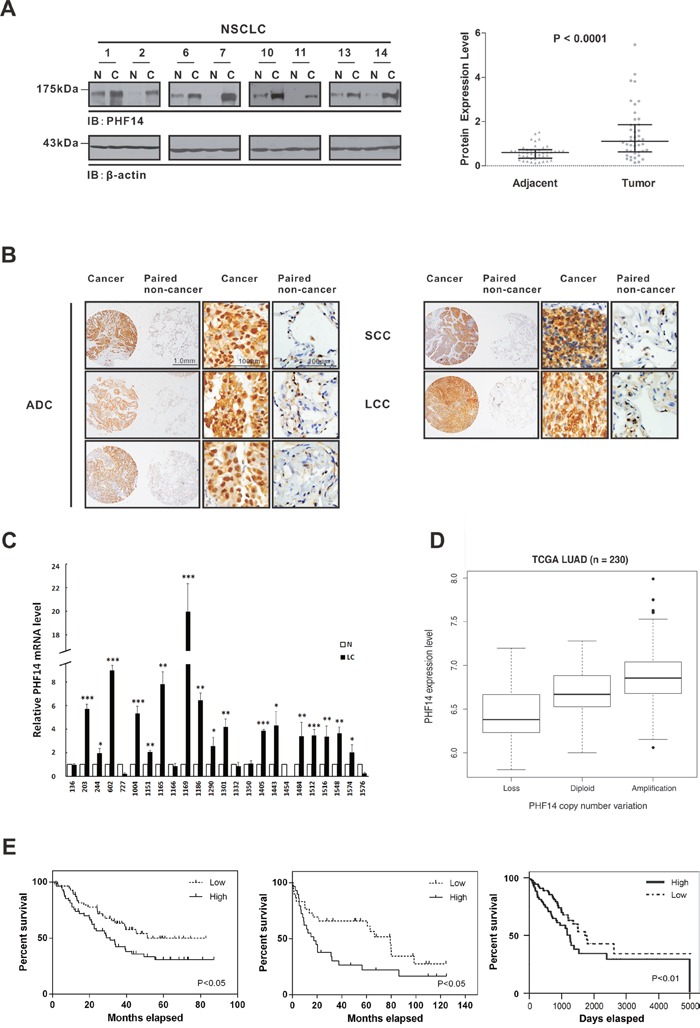
Upregulation of PHF14 in lung cancer is significantly associated with poor prognosis **A**. PHF14 expression in paired NSCLC samples (n=44). Left panel: representative images of western blots showing PHF14 expression in the NSCLC and adjacent tissues. β-actin was used for normalization. Right panel: statistical analysis of the relative PHF14 protein expression levels in the NSCLC and adjacent tissues. **B**. Representative images of immunohistochemical tissue arrays showing PHF14 expression in different types of NSCLC and adjacent tissues. ADC, adenocarcinoma; SCC, squamous cell carcinoma; LCC, large cell carcinoma. **C**. PHF14 mRNA levels in paired lung adenocarcinoma specimens (n=24). Differences between N (normal) and LC (lung cancer) were analyzed. **D**. Expression levels of PHF14 in lung adenocarcinoma are associated with gene copy numbers. Boxplot of PHF14 expression distribution in the sub-categorized adenocarcinoma patients based on PHF14 CNV (Copy Number Variation): Loss, Diploid and Amplification. The gene expression and CNV information was determined using RNA-seq and DNA-seq on the same cohort of 230 patients. Welch Two Sample t-test was used to compare the expression levels of PHF14 in subcategories. Amplification v.s. Diploid *P*=5.016e-06; Diploid v.s. Loss *P*=0.01473; Amplification v.s. Loss *P*=0.0003634. **E**. Kaplan-Meier survival curves of lung cancer patients corresponding to the PHF14 expression levels. The gene expression profiles were retrieved from GSE3141 (left panel, 111 samples) [34], GSE19188 (middle panel, 59 male subjects) [35] and TCGA database (right panel, 257 samples). The patients were categorized into two groups based on PHF14 expression level. “High” indicates that the expression level is higher than the samples’ median level; “Low” indicates lower. The log-rank test was used to compare the survival distributions of the two groups.

To study the potential role of PHF14 in tumors, we screened PHF14 expression in tumor tissues and their matched non-cancerous tissues from non-small cell lung cancer (NSCLC), hepatocellular carcinoma, colorectal carcinoma and renal cell carcinoma by western blot analysis. Interestingly, PHF14 expression was found to be strongly elevated in ca 80% (35/44) of NSCLC tissues with an average increase of 3-fold (Figure 1A and Supplementary Figure 1), while no obvious alterations of PHF14 expression in tumor tissues from hepatocellular carcinoma, colorectal carcinoma and renal cell carcinoma were observed (data not shown). To further verify this finding, additional 71 paired NSCLC samples were subjected to immunohistochemical analysis of tissue microarrays. About 82% (58/71) of tumor tissues exhibited a significant increase in PHF14 expression (score ≥9, up to 16, *P*<0.001) compared with paired non-cancer lung tissues using an immunoreactive scoring method (see Materials and Methods) (Figure 1B and Table 1). As shown in Figure 1B, the strong positive staining with purified anti-PHF14 antibodies (Supplementary Figure 2) was observed in tumor tissues, especially prominent in nucleus, while non-cancer tissues have little signal. In addition, qPCR analysis showed significant higher mRNA expression levels of PHF14 in 71% (17/24) of human lung cancer specimens (Figure 1C). Comparison of PHF14 expression in NSCLC and normal lung tissue samples from Gene Expression Omnibus (GEO) dataset GSE19188, the Cancer Genome Atlas (TCGA) NSCLC database or from our microarray data (GSE74095 and GSE74116) confirmed that PHF14 expression was upregulated at mRNA level in lung cancer samples (Supplementary Figure 1B-1D). The elevated mRNA levels are associated with increased gene copy numbers (Figure 1D).

**Table 1:** Relationship between the expression level of PHF14 and clinicopathological features of lung cancer

Variable	PHF14 expression (IRS)					*P*^a^ 8< score≤16 vs 1≤score≤8	*P*^a^ 12< score≤16 vs 1≤score≤12
	total	1≤score≤4	5≤score≤8	9≤score≤12	13≤score≤16		
		(−)	(+)	(++)	(+++)		
Sex							
male	43	3	7	17	16		
female	28	1	2	10	15	0.22	0.22
Age							
≤60	33	2	4	12	15		
>60	38	2	5	15	16	1	0.81
TNM stage							
I-II	53	2	6	22	23		
III	18	2	3	5	8	0.29	1
Pathological							
Non ADC	31	3	6	15	7		
ADC	40	1	3	12	24	0.06	P< 0.01
Metastasis							
positive	32	1	5	12	14		
negative	39	3	4	15	17	1	1
Tumor size							
≤3	47	3	5	21	18		
>3	24	1	4	6	13	0.75	0.22

Abbreviations: ADC, adenocarcinoma.

^a^ Calculated by fisher's exact test.

Analysis of the correlation between the overexpression of PHF14 and the clinicopathologic features of NSCLC revealed that high expression of PHF14 (score > 12) was correlated with adenocarcinoma (P<0.01) (Table 1). Interestingly, upregulation of PHF14 (score ≥ 9) were detected in a significant proportion (76%, 19/25) of early stage lung cancer patients (TNM I), which indicated its role in early carcinogenesis. In addition, 83% (39/47) of the lung cancer patients with small tumor size (≤3 cm) demonstrated PHF14 overexpression (Table 1). Furthermore, the high PHF14 expression level was significantly correlated with poor survival in lung cancer patients (Figure 1E). All of the above results suggest that PHF14 might be involved in the development of lung cancer.

### PHF14-knockdown significantly inhibits the proliferation of lung cancer cells

**Figure 2:**
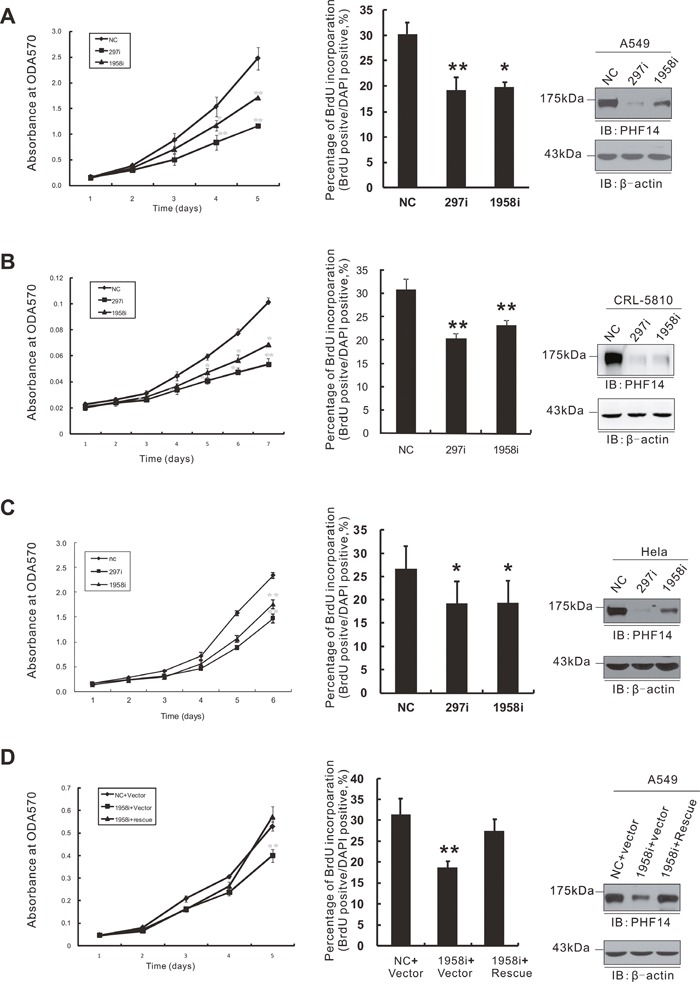
Knockdown of PHF14 inhibited cell proliferation Cell proliferation of PHF14-transiently knocked down A549 **A**., CRL-5810 **B**. and HeLa **C**. cells. **D**. Expression of siRNA-resistant PHF14 rescued the inhibitory effect of PHF14 RNAi. Left panels: results of the MTT assay performed in control cells and different PHF14-knockdown cells. Middle panels: statistical analysis of BrdU incorporation assay. Right panels: western blot analysis showing the efficiency of PHF14 knockdown and the expression level of siRNA-resistant PHF14 in the indicated cells. β-actin was used as a loading control. NC, non-targeting control siRNA-transfected cells; 297i, PHF14-297i siRNA-transfected cells; 1958i, PHF14-1958i siRNA-transfected cells. Data are shown as the mean values from three independent experiments ± s.d. (standard deviation). * P<0.05, ** P<0.01.

To investigate the possible role of PHF14 in lung cancer, we chose two lung adenocarcinoma epithelial cell lines with high expression of PHF14, A549 and CRL-5810 (Figure 5C), for further study. Two small interfering RNA fragments (siRNA/PHF14-297i and siRNA/PHF14-1958i) targeting human *PHF14* were transfected into lung cancer cells respectively and led to efficient suppression of endogenous PHF14 expression (Figure 2A and 2B, right panels). We subsequently monitored the proliferation of these cells up to one week using the MTT assays (Figure 2A and 2B, left panels). PHF14-depletion notably impaired the proliferation of A549 cells and CRL-5810 cells compared with non-targeting siRNA-transfected cells. We further confirmed the inhibitory effect of PHF14-depletion using Brd-U (5′-bromo-2′-deoxyuridine) incorporation assays (Figure 2A and 2B, middle panels) for detecting DNA synthesis. This inhibitory effect of the siRNAs could be rescued by exogenous expression of siRNA-resistant PHF14 in A549 cells (Figure 2D). In addition, RNAi of PHF14 also inhibited HeLa cell growth (Figure 2C). These results suggest a significant promoting role of PHF14 in cell proliferation of different cell lines.

### PHF14 depletion strongly suppresses the malignant transformation and tumorigenicity of NSCLC cells

**Figure 3:**
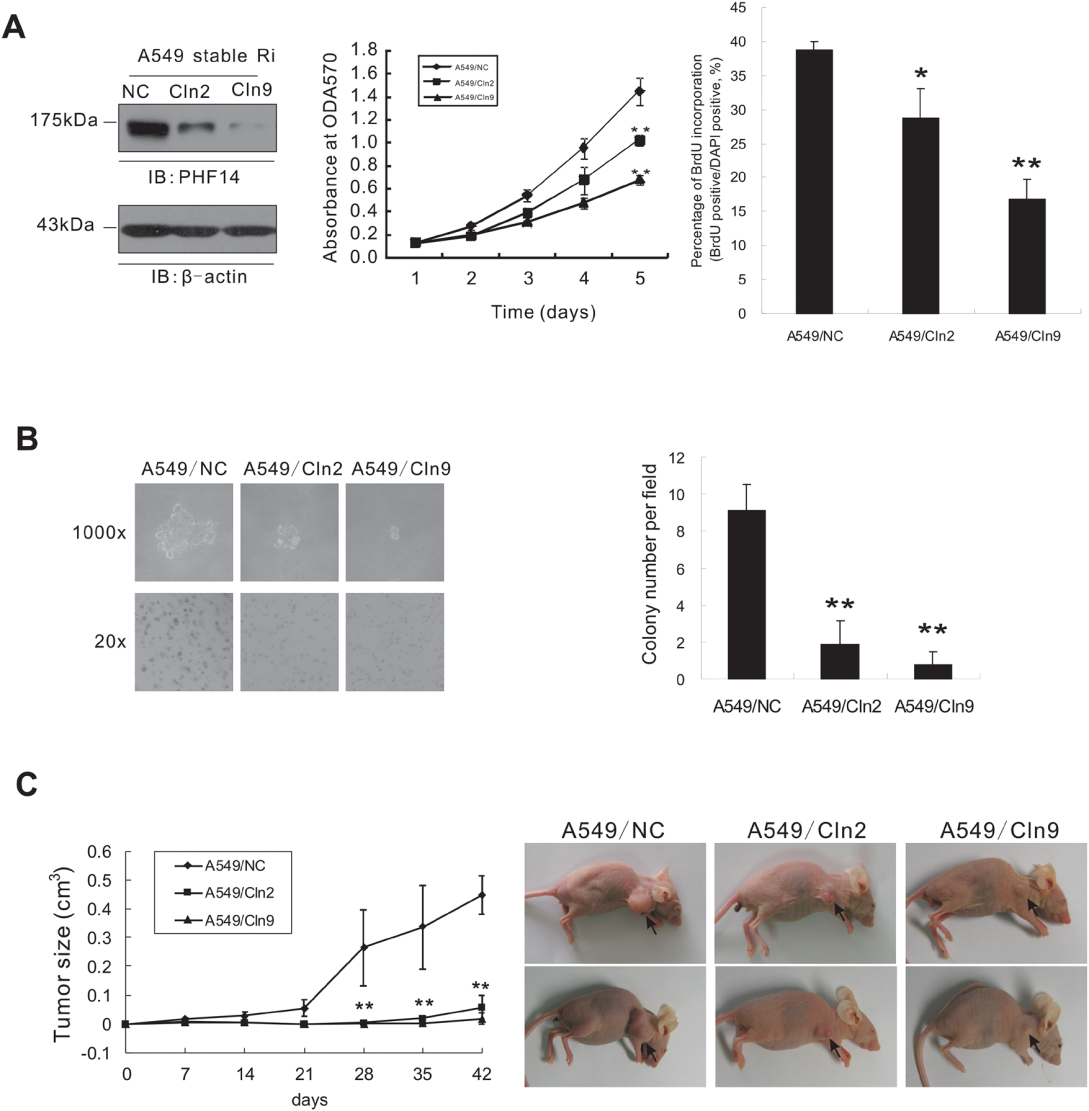
Stable knockdown of PHF14 in A549 cells inhibited tumorigenicity of lung cancer cells **A**. Cell proliferation of PHF14-stable knockdown A549 cells. Left panel: western blot analysis showing the efficiency of PHF14 knockdown in the indicated cells. β-actin was used as the loading control. Middle panel: results of the MTT assay performed in control cells and PHF14 stable knockdown clones. Right panel: BrdU incorporation assay. **B**. Soft agar assay. Cells were grown in soft agar culture medium for 15 days, and colonies (≥50 cells) were counted under a microscope. Left panel: representative images showing colony formation in soft agar. Right panel: colony numbers are presented as the mean value ± s.d. **C**. Tumor formation in nude mice. Left panel: Average tumor size in the mice injected with different cells as indicated. The mean value ± s.d. was calculated from four animals in each group. Right panel: representative images showing tumor formation in nude mice.* P<0.05, ** *P*<0.01.

To assess the effect of PHF14 on the malignant transformation of NSCLC cells, we performed an anchorage-independent growth assay. We established two transfectants, A549/Cln2 and A549/Cln9 originated from A549 cell lines, in which *PHF14* expression was stably silenced (Figure 3A) by expressing an shRNA construct targeting *PHF14* (derived from the siRNA equivalent to 1958i). As shown in Figure 3B, anchorage-independent growth of *PHF14*-knockdown tumor cells was strongly suppressed. A549/Cln2 and A549/Cln9 formed small pinpoint colonies consisting of less than 10 cells, whereas control cells (A549/NC pool) formed large colonies consisting of at least 50 cells. A statistical analysis of colony (≥50 cells) number revealed a significant difference between the control cells and the PHF14-knockdown cells (Figure 3B). To determine the effect of PHF14 on tumorigenicity *in vivo*, control A549/NC cells or PHF14-knockdown cells (pool cells, A549/Cln2 and A549/Cln9) were separately injected subcutaneously into nude mice. The control cells formed large tumors in nude mice. However, the knockdown of PHF14 dramatically reduced the ability of the cells to form tumors (Figure 3C & Supplementary Figure 3). Interestingly, the lower the PHF14 expression level, the slower the cells grew (Figure 3A), and the smaller the colonies (Figure 3B)/the tumors (Figure 3C) were. These data indicate that *PHF14* overexpression is critical for transformability and tumorigenicity of NSCLC cells.

### PHF14 directly interacts with KIF4A forming a physiological complex in the nucleus

**Figure 4:**
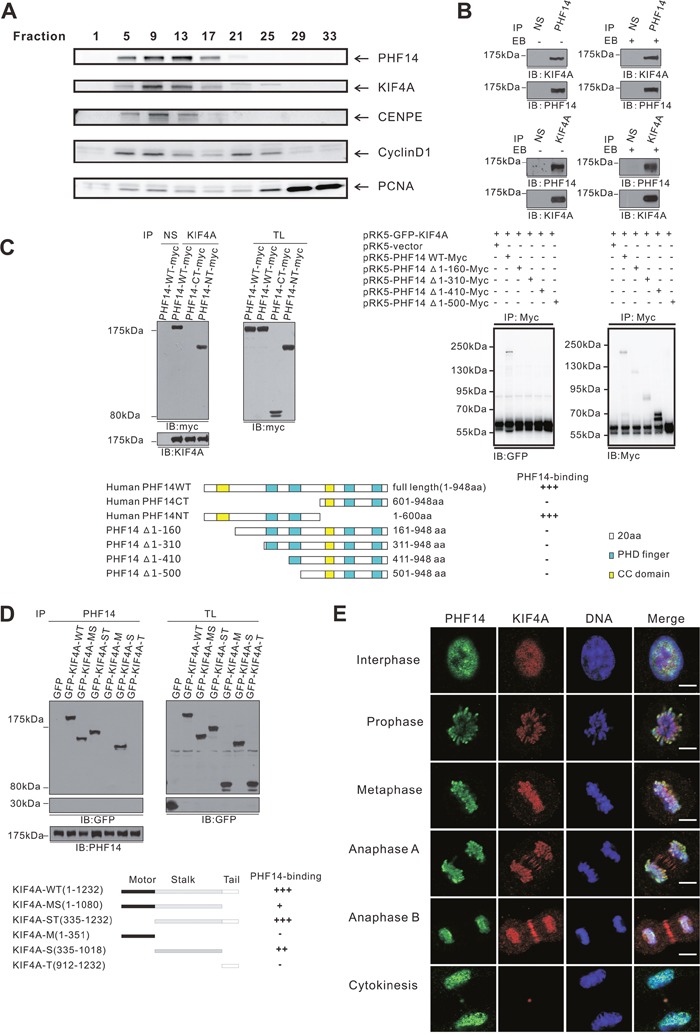
PHF14 and KIF4A formed a physical complex and co-localized extensively in the nucleus during mitosis **A**. FPLC strategy to identify protein complexes in nuclear extract from HeLa cells. PHF14, KIF4A, CENPE, and Cyclin D1 were detected in the same fractionations using western blotting analysis. **B**. Co-immunoprecipitation of endogenous PHF14 and KIF4A in A549 cells. **C**. and **D**. Mapping of the interaction site between PHF14 and KIF4A. Upper panels of (C): co-immunoprecipitation analysis of myc-tagged PHF14 or its mutants with GFP-tagged WT KIF4A. Myc-PHF14 or its mutants and GFP-KIF4A were co-overexpressed in HCT-116 cells. Lower panels of (C): schematic representation of PHF14 mutants. Upper panel of (D): co-immunoprecipitation analysis of GFP-tagged WT KIF4A or deletion mutants with PHF14. GFP-tagged WT KIF4A or deletion mutants were expressed in 293T cells. Lower panel of (D): schematic representation of KIF4Amutants. The affinity of the binding was rated as follows: +++: intensive; ++: moderate; +: weak; -: binding not detectable. **E**. Co-localization of endogenous PHF14 and KIF4A in A549 cells. A549 cells were stained with anti-PHF14 antibodies (green), anti-KIF4A antibodies (red) and DAPI (DNA, blue). Scale bar = 5 μm.

To further understand how PHF14 is involved in lung carcinogenesis, SILAC (Stable isotope labeling with amino acids in cell culture) proteomic approach was initially employed for analyzing its potential interaction partners. Numerous proteins were identified and can be categorized into multiple function groups including cell mitosis, cell proliferation, DNA repair, and epigenetic regulation (unpublished data). A group of nuclear proteins identified with high score, including centromere protein E (CENPE), cyclinD1, kinesin family member 4A (KIF4A) and proliferating cell nuclear antigen (PCNA) attracted our attention. These proteins have been implicated in cell mitosis and spindle assembly [18, 19]. To assess the potential functional association of PHF14 with those proteins, co-fractionation of nuclear proteins was performed by FPLC gel filtration. As shown in Figure 4A, KIF4A, CENPE and cyclinD1 were clearly co-fractionated with PHF14. Among them, only KIF4A was clearly co-precipitated with PHF14 in native A549 cells (Figure 4B). KIF4A has been reported to be a potential marker in lung cancer [17] and to play critical roles in multiple aspects of mitotic and chromatin regulation [19–21]. This interaction was further confirmed by reciprocal immunoprecipitation with anti-KIF4A antibodies (Figure 4B). We further mapped the binding regions between PHF14 and KIF4A to the N-terminal region of PHF14 1-160 a.a which contains a coiled-coil domain and the stalk domain of KIF4A, respectively (Figure 4C, 4D, and Supplementary Figure 4). Moreover, immunostaining analysis demonstrated that PHF14 and KIF4A were co-localized not only in the nuclei in interphase cells but also dynamically on the condensed chromatin in mitotic cells at different stages (Figure 4E). These data suggest that PHF14 binds to KIF4A to form a novel functional complex in the nucleus, which may play a role in cell mitosis.

**Figure 5:**
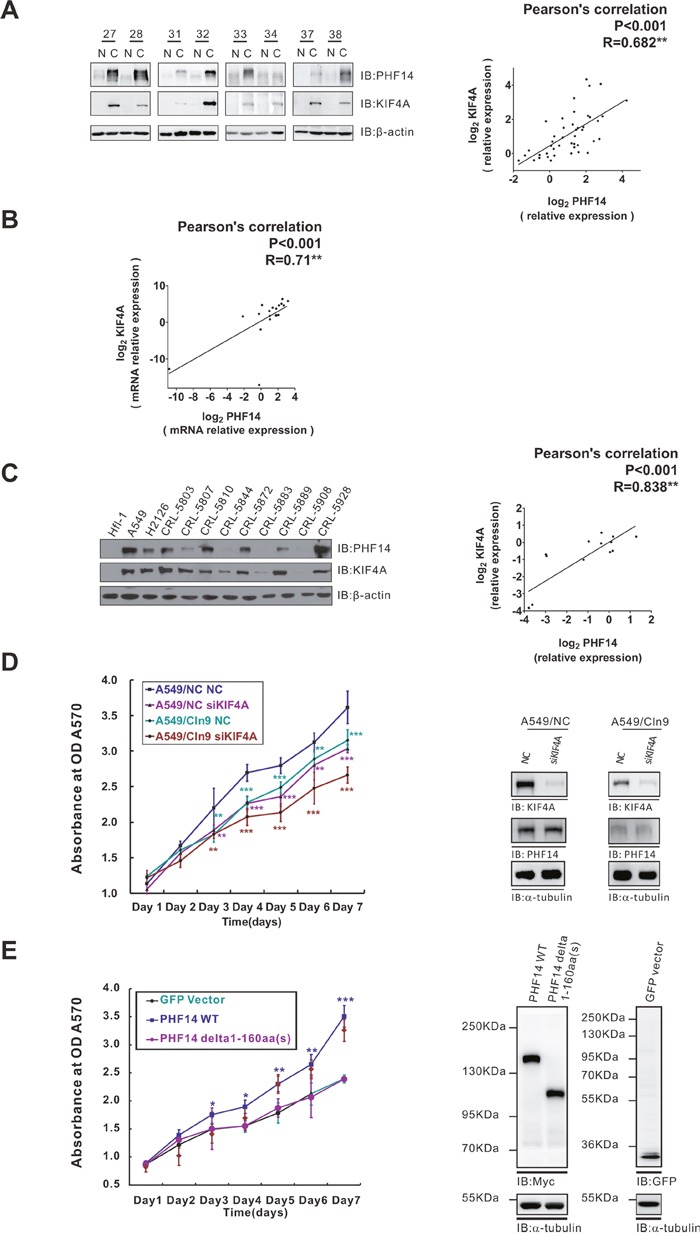
PHF14 co-overexpressed with KIF4A and they have a synergistic effect on cell proliferation **A**. PHF14 and KIF4A expression in paired NSCLC samples. Left panel: representative western blot results showing that PHF14 and KIF4A are co-upregulated in NSCLC specimens. β-actin was used as a loading control. Right panel: Pearson's correlation analysis showing a significant positive correlation between PHF14 expression and KIF4A expression in NSCLC specimens (n=44). R=0.682, *P*<0.001. **B**. The correlation between PHF14 and KIF4A mRNA expression levels in paired NSCLC samples. Data obtained from qPCR results, n=24, R=0.71, *P*<0.001. **C**. PHF14 and KIF4A expression in NSCLC cell lines. Left panel: western blot results showing that PHF14 and KIF4A are co-upregulated in NSCLC cell lines. A human fetal lung fibroblast (HFL) was used as a control. β-actin was used as a loading control. Right panel: significant positive correlation between PHF14 expression and KIF4A expression in NSCLC cells. R=0.838, *P*<0.001. **D**. The effect of PHF14 and/or KIF4A depletion on A549 cell proliferation. **E**. The effect of overexpressing PHF14 WT or PHF14 NT 1-160a.a truncated mutant on HCT-116 cell proliferation. Left panel: results of the MTT assay. Right panels: western blot analysis showing the knockdown efficiency of different siRNAs and efficiency of the overexpression of different constructs. α-tubulin was used as the loading control. * *P*<0.05, ** *P*<0.01, *** *P*<0.001.

### PHF14 and KIF4A are co-overexpressed and co-activated in lung cancer

KIF4A was reported to be overexpressed and highly activated in NSCLC samples [17]. Indeed, KIF4A was overexpressed in 73% (32/44) of our NSCLC samples (Figure 5A), which was similar with previous data [17]. We noted that the expression patterns of PHF14 and KIF4A in these lung cancer samples are quite the same (Figure 5A, Supplementary Figure 1). The correlation analysis demonstrated that the co-overexpression of KIF4A and PHF14 had a significant positive correlation at both the protein (R = 0.682, P < 0.001, Figure 5A) and mRNA levels (R = 0.71, *P* < 0.001, Figure 5B). This co-overexpression pattern of PHF14 and KIF4A were also detected in randomly picked 11 human NSCLC lines when a normal fetal lung fibroblast cell line HFL-1 was used as control (R = 0.838, *P* < 0.001, Figure 5C). To confirm this observation, we analyzed the correlation between PHF14 and KIF4A expression in a much larger scale (Supplementary Figure 5, n=516). Again, the two genes showed a positive correlation in their expression (R=0.246, *P* < 0.001).

Next, we sought to investigate the role of the PHF14-KIF4A complex in co-regulating cell proliferation. KIF4A and/or PHF14 were knocked down in A549 cells. PHF14- or KIF4A-depletion alone notably impaired the proliferation of the cancer cells. Double-depletion of the two molecules resulted in a significant synergic effect on further inhibiting tumor cell proliferation (Figure 5D). To further prove the importance of PHF14-KIF4A binding in tumor cell proliferation, HCT-116, a colon carcinoma cell line lacking endogenous expression of PHF14 [15], was employed. As expected, overexpressing wild type PHF14 but not the KIF4A-unbound PHF14 mutant (Δ1-160a.a) in HCT-116 strongly promoted cell proliferation (Figure 5E). These results suggested an essential role of the KIF4A-binding domain in promoting cell proliferation.

### PHF14 cooperates with KIF4A to regulate mitosis

**Figure 6:**
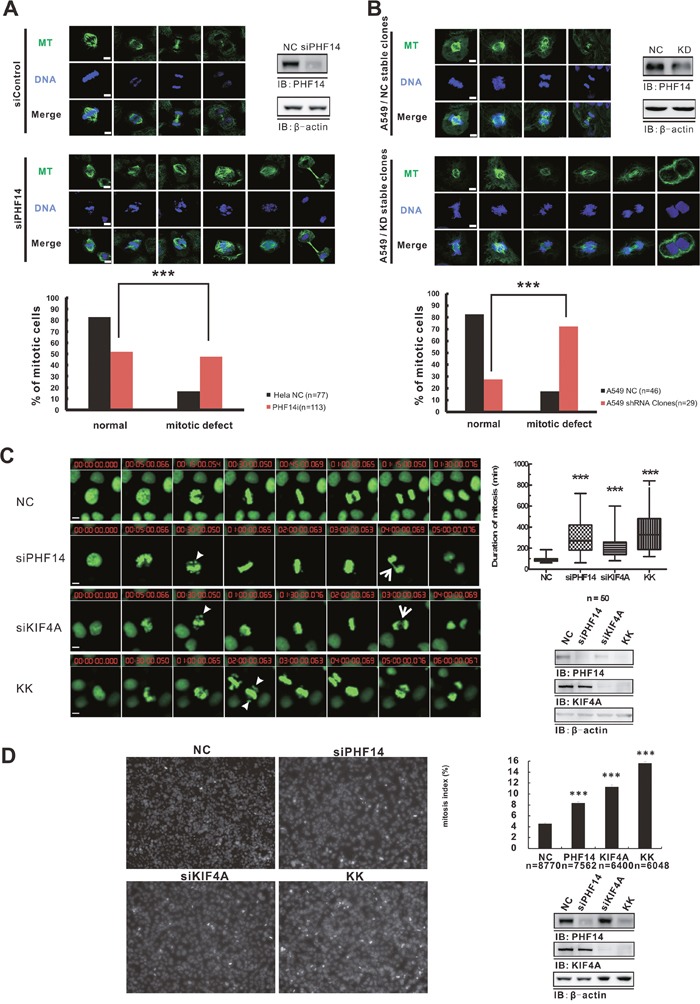
Depletion of PHF14 and KIF4A induced mitotic defects Chromosome and spindle morphology defects during mitosis in PHF14-depleted HeLa cells A. and PHF14-depleted A549 cells B. Upper panels: cells were stained with anti-tubulin (green) and DAPI (DNA, blue) to reveal the spindle and the chromosome. Chromosome misalignment, anaphase separation incompletion, and spindle defects of the distinct mitotic cells can be seen in PHF14-depleted cells. Bar, 5μm. Lower panels: statistics of mitotic phenotypes. C. The effect of PHF14 and/or KIF4A depletion on mitosis in HeLa cells. Left panel: representative images from time-lapse movies. HeLa cells stably expressing histone H_2_B-GFP (green) were transfected with control siRNA (NC), PHF14 siRNA (siPHF14), KIF4A siRNA (siKIF4A) or PHF14/KIF4A siRNA (KK). Bar, 5 μm. Arrowheads indicate mis-aligned chromosomes and arrows indicate incomplete-separated chromosomes inPHF14RNAi and/or KIF4A RNAi cells. Upper right panel: quantitative analysis of the duration of the M phases of HeLa H_2_B-GFP cells after different siRNA treatments. Data from at least three different experiments are represented as box-and-whisker plots. D. Analysis of mitotic index in HeLa cells. Left panel: representative cell images of HeLa cells transfected with different siRNAs. Random images (9 images, 6,000–8,000 cells) were analyzed per experiment, and mitotic cells were identified in the 405 nm channel on the basis of their condensed DNA content. Upper right panel: the mitotic index was calculated and is presented as the mean ± s.d.; *** *P*<0.0001. n=number of analyzed cells. Lower right panels: western blot results showing the efficiency of PHF14 and/or KIF4A knockdown in the indicated cells. β-actin was used as the loading control.

KIF4A is an important component of the chromosome condensation and segregation machinery functioning in multiple steps of mitotic division and plays essential roles in regulating anaphase spindle dynamics and the completion of cytokinesis [19–21]. In addition to the co-overexpression in lung cancer cells, PHF14 and KIF4A form a functional complex in native cells (Figure 4E). These findings promoted us to further investigate whether PHF14 has effect on cell mitosis. Knocking down PHF14 in HeLa cells and A549 cells clearly exhibited multiple severe mitotic defects including misaligned chromosomes, spindle disorganization, and defective cytokinesis (Figure 6A and 6B). Quite interestingly, PHF14-depeltion induced abnormalities were very similar to those caused by losing KIF4A (Figure 6C) [21]. Together, our results suggested a critical role of PHF14 in chromosome alignment and separation, spindle organization, and cytokinesis.

Mitotic defects usually indicate an abnormal M phase. We further explored the effect of PHF14 and KIF4A on the cell cycle. Using siRNAs to knockdown PHF14 or KIF4A resulted in significantly prolonged M phase in H_2_B-GFP overexpressing HeLa cells, while knocking down both had the strongest effect (Figure 6C and Supplementary Movie 1–4). The duration of the mitotic phase increased from 89.70 ± 3.268 min in control cells to 303.5 ± 23.98 min in PHF14-knockdown cells, to 214.9 ± 15.50 min in KIF4A-knockdown cells and to 369.4 ± 29.26 min in double knockdown cells (Figure 6C). The mitotic index also increased around 2-fold in the case of losing PHF14 or KIF4A (Figure 6D), which suggested mitotic arrest. The index increased to almost 4-fold when the both proteins were knocked down in HeLa cells (Figure 6D). This is consistent with the prolonged M phase in these cells (Figure 6C). All these data clearly suggested that PHF14 and KIF4A played a co-operative role in mitosis.

## DISCUSSION

PHD proteins can function as oncogenic proteins [22, 23]. PHF14 is a newly identified PHD finger protein with unclear biological functions. Here, we found that PHF14 was co-overexpressed with KIF4A in lung cancers and the two proteins formed a functional complex involved in cell mitosis and tumorigenesis.

Several independent cancer cohorts were recruited for studying the significance of PHF14 in carcinogenesis. Using various analyses, we found that more than 70% lung cancer tissues exhibited overexpression of PHF14, which was significantly associated with shorter survival in lung cancer patients. Interestingly, high expression of PHF14 can be detected not only in advanced lung cancer patients, but also at early stage (TNM I) and in small size tumor (≤3cm). This may indicate that PHF14 plays an early and long-lasting role throughout the entire process of carcinogenesis. The high expression level of PHF14 in early cancer samples makes it a promising early diagnostic marker in lung cancer. The enhanced signal of PHF14 in cancer tissues is more prominent in nucleus, which is consistent with its potential role in epigenetic regulation [12] and mitosis (Figure 6). Intriguingly, in some cases, strong cytoplasmic signal can be detected. This may indicate other unidentified functions of PHF14/ different isoforms [12] or mutations of PHF14 (Supplementary Table 1 & 2) in lung cancers. Knockdown of PHF14 in cancer cells strongly inhibited cell proliferation, transformation, and tumor formation in nude mice, supporting an important function of PHF14 in promoting the carcinogenesis of lung cancer. Notably, increased knockdown efficiency of PHF14 resulted in more pronounced inhibitory effects. This is consistent with the observation that higher PHF14 expression is associated with poorer prognosis. So far, pronounced dysregulation of PHF14 has only been found in lung cancer. Its absence in a colon cancer cell line [15] and a biliary tract cancer cell line [16] may suggest PHF14 plays different roles in different cells/cancer types. As the expression level of PHF14 in colon cancer/biliary tract cancer patients is not clear, the clinical relevance of these findings remain to be elucidated. This is the first time that an important role of PHF14 in carcinogenesis and its clinicopathological significance have been reported.

To understand the underlying molecular mechanisms, we tried to identify the potential interaction partners of PHF14. KIF4A, a potential marker for lung cancer [17], is detected by mass spectrometry with high abundance (unpublished data). KIF4A is a microtubule-based motor protein playing key roles in chromosome integrity during mitosis, the organization of the spindle, midzone formation, and cytokinesis [24–26, 29, 30]. It has also been implicated as an important molecule involved in DNA damage response and/or DNA repair, possibly through poly (ADP-ribose) polymerase-1 (PARP-1) and the breast cancer 2 (BRCA2) pathway [35, 36]. Interestingly, several potential PHF14-associated proteins with high hits (including PARP-1) in our proteomics analysis also have functions related to DNA repair and cell mitosis (unpublished data). And similar to PHF14, KIF4A has been related to poor prognosis in lung cancers [17]. Besides their functional relevance, both proteins were co-localized on chromatin throughout the cell cycle, forming a complex in cells. Co-overexpression of PHF14 and KIF4A was detected at both protein and mRNA levels, strongly suggesting a functional connection between the two proteins. Depletion of PHF14 and KIF4A had additive effects on the mitotic index and cell proliferation. The synergistic effects of these two molecules were further confirmed by the failure of KIF4A-unbound PHF14 mutant to promote cell proliferation. Thus, we propose that the two proteins in cancer cells form a complex to work together in tumorigenesis. Interestingly, stable knockdown of PHF14 in lung cancer cells seems to cause a downregulation of KIF4A (Supplementary Figure 3B), which may indicate an intrinsic functional regulation between the two proteins. Yet, using siRNA knockdown approach, we found no clear evidence that PHF14/KIF4A reciprocally regulates each other's expression (data not shown). Those data implies that the effect of PHF14 on KIF4A expression may be indirect as functional synergistic regulation; or the co-overexpression of the two proteins is regulated by some common regulator(s). The detailed interaction between the two molecules is currently under investigation. Both KIF4A and its binding proteins such as PARP-1, BRCA2 and PRC1 have been implicated in multiple types of human cancers [27–32]. It will be of great interest to recruit more patients with different cancer types to test whether PHF14 and KIF4A are co-overexpressed and co-activated in other cancer types.

PHF14/KIF4A complex may promote tumorigenesis in two ways: increasing mitotic defects and DNA errors. On one hand, mitotic defects resulted in a prolonged M phase, which may explain why depletion of PHF14 and/or KIF4A inhibited cell proliferation. On the other hand, mitotic errors and DNA-level defects are often associated with genomic instability which contributes significantly to tumor progression [33]. Interestingly, PHF14 depletion also hampered DNA repair (unpublished data). What remains to be clarified is the downstream target proteins of PHF14/KIF4A and their detailed mechanism in regulation mitosis/DNA repair.

The functions of PHF14, as PHD finger protein, may not be limited to mitotic regulation. Epigenetic alteration is a common event in cancers and significantly contributes to the neoplastic phenotype. A well-known example is the ING family [8]. All of the four PHD domains of PHF14 are conserved from Drosophila to humans, thus underscoring their importance. PHF14 has been reported to act as a transcription factor or a member of a transcription factor complex and to suppress PDGFRα expression in mesenchymal cells [13]. Whether PHF14 can regulate the expression of oncogenes/tumor suppressors, thereby leading to carcinogenesis, is intriguing and should be investigated.

In summary, PHF14 is a novel regulator of mitosis whose upregulation is significantly related to NSCLC. PHF14 may cooperate with KIF4A in mitotic control or cell proliferation regulation and thus contribute to lung carcinogenesis. The novel PHF14/KIF4A complex might indicate a fundamental interdependent role for PHF14 and KIF4A in the carcinogenesis of lung cancer. Our data provide new insights into the biological significance of PHD finger proteins and also imply that PHF14 or PHF14/KIF4A complex are potential biomarkers for diagnosis and treatment of NSCLC.

## MATERIALS AND METHODS

### Plasmids

The PHF14 deletion mutants were constructed from the full-length human PHF14 construct [12] and subcloned into pRK5-R_S_-myc or pRK5-R_S_-GFP using the *Eco*RI and *Xba*I sites. The fragment encoding PHF14 1-160a.a was subcloned into pGEX-5×1 using the *Eco*RI and *X*hoI sites. The full-length human KIF4A ORF and The fragment encoding KIF4A 858-1232 a.a in pGEX-5×1 were kind gifts from Professor Wei Jiang (Sanford Burnham Institute). KIF4A deletion mutants were subcloned into pEGFP-C1 using the BglII and SalI sites. All the plasmid constructs were verified by DNA sequencing. For overexpression of exogenous plasmid, Lipofectamine® LTX & Plus Reagent (Invitrogen, USA) were used according to the manufacturer's instructions.

### Antibodies

The anti-PHF14 (against GST-fused PHF14 1-160 a.a.), anti-KIF4A (against GST-fused KIF4A 858-1232a.a), and anti-GST rabbit/mouse polyclonal antibodies were produced in our own laboratory. All the other antibodies used in this study were commercial antibodies: anti-β-actin, anti-α-tubulin (Sigma, USA); anti-GFP (Abcam, UK); anti-Myc (Merck Millipore, Germany).

### Silencing of genes

For silencing of PHF14 by small interfering RNA, two siRNAs targeting positions 297-315 (5′-GGAAAAGAAGGAAGAAGAA-3′; named PHF14-297i) and 1958-1976 (5′-AGCUUCAUGUAGAAU AUAA-3′; named PHF14-1958i) of the human PHF14 ORF were selected. A non-targeting control siRNA (5′-UUCUCCGAACGUGUCACGU-3′) was used as a negative control (NC). Primer 5′-GAATGAACAAGAAAAGCTGCATGTAGAGTATAATAAGCTATGTGAATC-3′ was used to construct PHF14 siRNA (1958i)-resistant construct. For silencing of KIF4A, siKIF4A (5′-GCAAGAUCCUGAAAGAGAUTT-3′) was used according to the previous report [21]. The siRNAs were transiently transfected into A549 or CRL-5810 cells using Lipofectamine^®^ RNAiMAX Transfection Reagent from Invitrogen according to the manufacturer's instructions.

### Retrovirus production and stable clone selection

The polynucleotide of PHF14-1958i or NC was synthesized and inserted into the RNAi-Ready pSIREN-RetroQ vector (Clontech, USA) according to the manufacturer's instructions. The pcL10A1 plasmid was co-transfected with pSIREN-RetroQ-NC or pSIREN-RetroQ-1958i into 293T cells. Lipofectamine® 2000 Transfection Reagent from Invitrogen was used following the manufacturer's instructions. Forty-eight hours later, the medium was collected and passed through a 0.45-μm filter to get the viral stock. The viral stock was added to A549 cells. Twenty-four hours post-infection, puromycin was used to select transfected cells.

### Cell lines and specimen collection

The lung cancer cell lines used in this study (except for CRL-5810, CRL-5844 and CRL-5844) are kind gifts from Professor Axel Ullrich (Martinsried, Germany). The human lung cancer cells were maintained in RPMI 1640 from Invitrogen. All the other cells used in this study were cultured in DMEM from Invitrogen. Culture media were supplemented with 10% fetal bovine serum (FBS) from Invitrogen.

A tissue microarray for the immunostaining analysis of PHF14 protein expression was purchased from Shanghai Outdo Biotech (China). The array consisted of 71 paired NSCLC cancer and matched non-cancer tissues and four normal lung tissues. For qPCR analysis, 24 paired NSCLC samples were collected from Fudan University Shanghai Cancer Center. For western blot analysis, 24 paired NSCLC samples were obtained from Changhai Hospital affiliated to the Second Military Medical University, additional 20 paired NSCLC samples were obtained from Chest Hospital affiliated to Shanghai Jiaotong University. The access to human tissues complied with both Chinese laws and the guidelines of the local ethics committee. All patients participating in this study gave informed consent prior to surgery and data collection.

### Quantitative real-time polymerase chain reaction (qRT-PCR)

NSCLC samples were harvested in Trizol (Invitrogen) for total RNA extraction, and RNA was reverse-transcripted by PrimeScript RT reagent Kit with gDNA Eraser (TaKaRa, Japan). Quantitative real-time PCR analyses were carried out with SYBR Green Master (ROX, Roche) on ABI 7500 fast thermal cycler and analyzed with 7500 Software v2.0.1. Expression levels are normalized to β-actin (*ATCB*) and presented relative to that of paired normal samples. PCR oligo sequences are listed as follows: for *PHF14*, 5′- CGGATGGATATGTCAGGAATG-3′and 5′-TGTTCCATGTTGAGCTCCTG-3′; for *KIF4A*, 5′- TACTGCGGTGGAGCAAGAAG-3′ and 5′- CATCTGCGCTTGACGGAGAG-3′; for *ATCB*, 5′- ACCGCGAGAAGATGACCCAG -3′ and 5′- CCATCTCGTTCTCGAAGTCCA -3′.

### Immunoprecipitation and western blot analysis

Immunoprecipitation assay was performed as described before [12]. To avoid chromatin-mediated protein interactions, ethidium bromide (EB) was added in IP buffer during the incubation. Mice tissue was homogenized using Tissuelyser (Tissuelyser-24, Shanghai Jingxing Experimental technology) in high salt lysis buffer (1% NP-40, 0.5M NaCl,10% sucrose, 40mM Tris-HCl pH 7.5, protease inhibitor cocktail). BCA protein assay kit (Tiangen, China) was used for protein quantification. Western blotting was performed according to conventional methods. The western blots were quantified using Quantity One software (Bio-Rad Laboratories Inc., USA).

### Co-fractionation of nuclear proteins by fast protein liquid chromatography (FPLC)

HeLa cell nuclei were lysed in RIPA buffer and separated by conventional gel filtration. An AKTA Protein Purification System was used in this experiment. The nuclear extracts in buffer (50 mM Tris-HCl pH8.0, 0.3 M NaCl, 1 mM EDTA) were applied on a HiLoad 16/60 Superdex 200 column from GE Healthcare. Proteins were eluted at 1 ml/min flow rate using PBS. The fractions were collected and analyzed by western blotting.

### Immunofluorescence

Cells grown on coverslips (Fisher Scientific, USA) were fixed with methanol at -20°C for 3-5 min. The cells were subsequently incubated with the primary antibodies, Alexa Fluor® 546-conjugated /Alexa Fluor® 488-conjugated goat anti-rabbit/mouse IgG (Thermo Fisher Scientific, USA), 4′, 6-diamidino-2-phenylindole (DAPI) from Sigma, and were mounted using PERMAFLUOR aqueous mounting medium (Immunotech, France). Samples were analyzed using laser-scanning confocal microscopy (Leica, TCS SP5, Germany).

### Immunohistochemistry analysis and immunoreactive score (IRS)

Tissues were processed into paraformaldehyde-fixed, paraffin-embedded specimens. Sections were incubated with anti-PHF14 (produced in our own laboratory) followed by biotinylated secondary antibodies and peroxidase-conjugated streptavidin (Thermo Fisher Scientific). Finally, the sections were counterstained with hematoxylin and examined. Positivity for PHF14 positive cells and staining intensity was assessed semi-quantitatively by three independent investigators without prior knowledge of the clinical follow-up data. Cases were accepted as positive only if reviewers independently defined them as such. The percentage of positive cells was rated as follows: 1, 1-10% positive cells; 2, 11-50% positive cells; 3, 51-75% positive cells; and 4, >75% positive cells. Staining intensity was scored as follows: 1, negative; 2, weak; 3, moderate; and 4, intensive. The scores for the percentage of positive cells and the scores for expression intensity were multiplied to calculate an immunoreactive score (IRS): 1-4 +/−; 5-8 = weak staining; 9-12 = moderate staining; and 13-16 = strong staining.

### Cell proliferation assay

After being cultured for the indicted amount of time, the cells were incubated with 3-(4, 5)-dimethylthiazol (−zyl)-3,5-diphenyltetrazolium bromide (MTT; 5 mg/ml) for 4 hrs. The cells were then incubated with dimethyl sulfoxide (DMSO) in the dark. The color intensity was quantified by measuring the absorbance at 570 nm using a microplate reader from Bio-Rad Laboratories Inc. DMSO alone was used as a control.

For the 5-bromo-2′-deoxyuridine (BrdU) incorporation assay, the cells were grown on glass coverslips to 50% confluence. BrdU was added, and the cells were cultured for another 0.5 h. The cells were then fixed and stained with a BrdU antibody and DAPI using the BrdU labeling and detection kit I (Roche, Germany). At least 1000 cells were counted per group.

### Colony formation in soft agar

A 2-mL portion of 0.6% agar from Sigma in DMEM supplemented with 10% FBS was added into 6-well plates and allowed to harden. Subsequently, 0.5 × 10^3^ cells in DMEM supplemented with 10% FBS and 0.3 % agar were seeded on the polymerized agar. The cell solution was allowed to set for 30 min at room temperature before being moved into a 37°C, 5% CO_2_ incubator. Cell colonies (≥ 50 cells) formed in soft agar were counted and photographed 15 days after inoculation. Nine fields were counted for each cell line.

### *In vivo* tumorigenicity assay

For each cell line, 4 × 10^6^ cells were inoculated subcutaneously into the right shoulder area of male BALB/c nude mice. Tumor formation was monitored weekly by measuring the largest and the smallest diameter of the formed tumors, and the volume of the tumors was calculated using the following formula: volume = 1/2 × (largest diameter) × (smallest diameter)^2^. The experiments were conducted in accordance with the guidelines for care and use of laboratory animals and approved by Animal Care and Use Committee of SIBCB.

### Live-cell imaging

HeLa cells stably expressing histone H_2_B-GFP were a kind gift from Prof. Xueliang Zhu (Shanghai Institutes for Biological Sciences, China). Groups of cells were transfected with different siRNAs, and time-lapse movies were acquired 48 hours after transfection using the UltraVIEWVoX 3D Live Cell Imaging System (PerkinElmer Inc., USA). Mitotic cells were selected (on the basis of their histone H_2_B-GFP fluorescence) and imaged during mitosis. Images were captured every 5 min over a period of 18-24 hrs. For each group, 3-5 randomly selected fields were acquired.

### Mitotic index

HeLa cells were transfected with each siRNA, fixed, and stained with DAPI 48 hrs after transfection. Cell images were acquired using a high content analysis system Operetta from PerkinElmer Inc. using a 20x objective. The Harmony software was used to analyze the large volume of data. The mitotic index was calculated using the following formula: cells observed with visible chromosomes/total number of cells visible×100%.

### Clinical and statistical analysis

The gene expression profiles were retrieved from two previous reports (GSE3141 [34] & GSE19188 [35]) and The Cancer Genome Atlas (TCGA) https://cancergenome.nih.gov/. The Statistical Package for the Social Sciences (SPSS) and Microsoft Excel were used for statistical analysis. Student's t test was used (unless mentioned elsewhere) based on the assumption that the distribution of the data follows normal distribution. The data are presented as the mean value± s.d. All experiments were repeated at least three times. Differences between experimental and control groups (usually NC) were considered significant if P-values were <0.05 (*), 0.01 (**) and 0.001 (***). Pearson's rank Correlation Analysis and the Kaplan-Meier plot of survival analysis were performed with SPSS 16.0 software (SPSS Inc., 1989-2007, USA). All of the Fisher's Exact Tests were analyzed on the website http://www.langsrud.com/fisher.htm

## SUPPLEMENTARY MATERIALS FIGURES AND TABLES










