# Supplementary material for: A novel PHD-finger protein 14/KIF4A complex overexpressed in lung cancer is involved in cell mitosis regulation and tumorigenesis
Source: Oncotarget. 2017 Feb 1;8(12):19684–98. doi: 10.18632/oncotarget.14962 (PMC5386714; doi:10.18632/oncotarget.14962)
Supplement: Supplementary file 1 [file oncotarget-08-19684-s001.pdf]

A novel PHD-finger protein 14/KIF4A complex overexpressed in lung cancer is involved in cell mitosis regulation and tumorigenesis

SUPPLEMENTARY DATA

SUPPLEMENTARY METHODS

In vitro binding assay

GST-PHF14 (N-terminal 1-160 a.a) and GST were purified from BL21 bacteria and eluted from glutathione-Sepharose 4B beads (GE Healthcare, UK) as suggested by the manufacturer. For binding assays, pEGFP-C1 KIF4A full length was incubated with equivalent amounts of GST or GST-PHF14 (N-terminal 1-160 a.a) fusion proteins. After incubation, bead-bound proteins were separated by SDS-PAGE and analyzed by western blotting.

SUPPLEMENTARY FIGURES AND TABLES

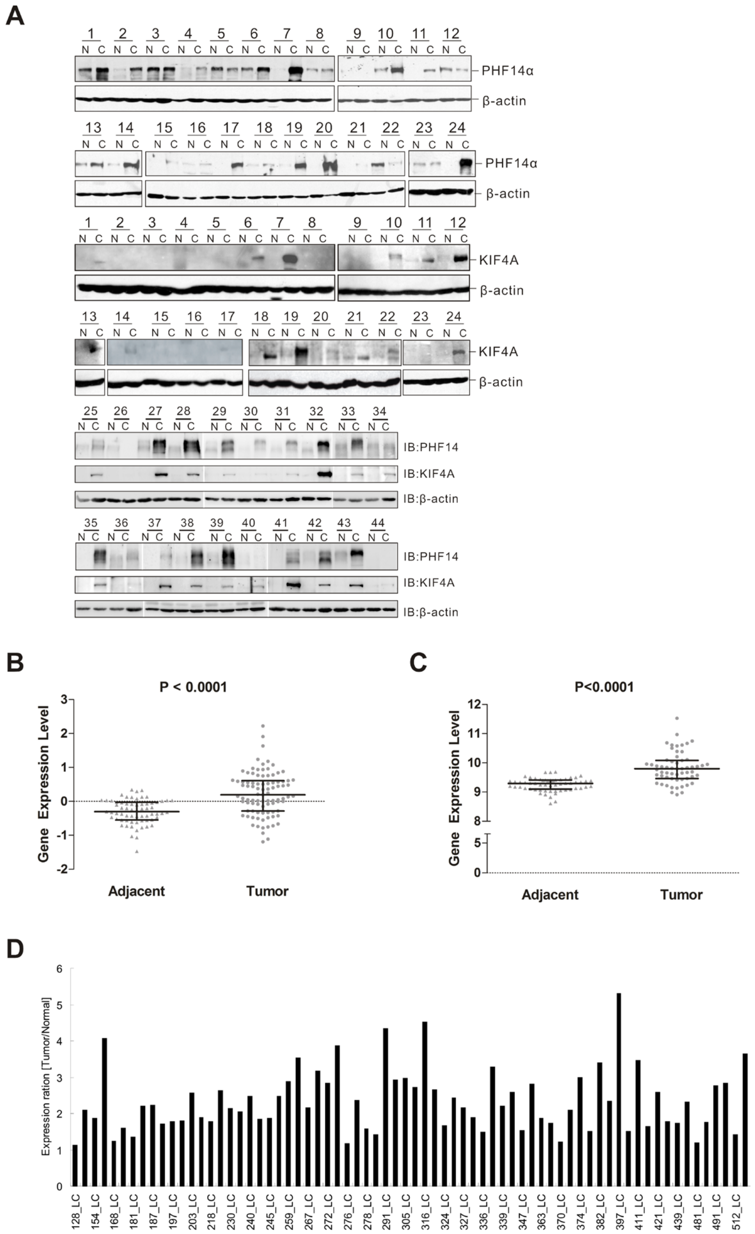

Supplementary Figure 1: PHF14 and KIF4A are upregulated in lung cancer. A. PHF14 and KIF4A expression in NSCLC (n=44). Images of western blot showing PHF14 expression in the cancer and adjacent tissues. β-actin was used for loading control. **B.** The boxplot of PHF14 gene expression levels in 91 tumor and 65 adjacent normal lung tissue samples. The gene expression profiles were retrieved from dataset GSE19188 (NCBI GEO accession number), probeset identifier 228095_at. **C.** Statistical analysis of the PHF14 mRNA expression levels in the lung adenocarcinoma and adjacent tissues. The RNAseq gene expression profiles were retrieved from TCGA database (n=58). For B & C: The first and the third lines indicate the first and third quartiles, respectively; the second line is the median. Student's t-test was used to compare the gene expression levels from the two groups. **D.** PHF14 mRNA levels in lung adenocarcinoma specimens from microarray data (GSE74095 with 78 Lung ADC samples and GSE74116 with 10 paired normal lung samples). Average values obtained from all probesets used in this assay for PHF14 were presented. PHF14 expression value in each tumor sample was calculated against the mean value in normal samples.

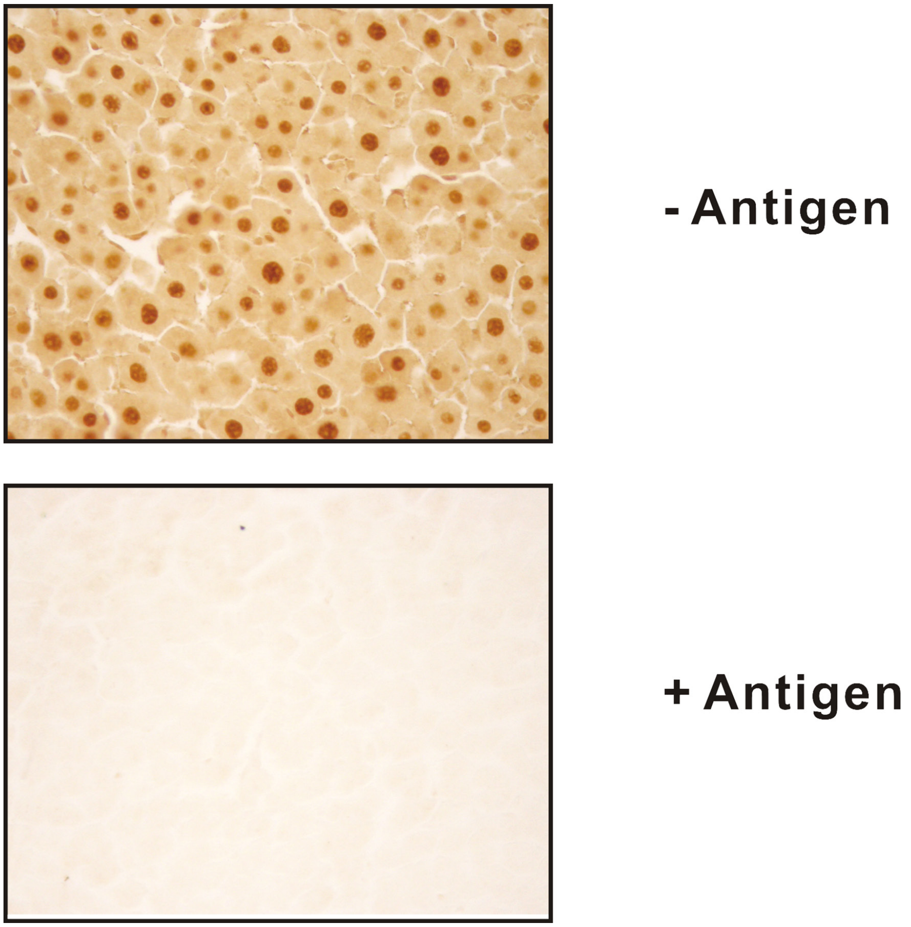

Supplementary Figure 2: The specificity of the PHF14 polyclonal antibody. Immunohistochemical analysis of mouse liver showing PHF14 expression using the anti-PHF14 polyclonal antibody without (upper) or with (bottom) pre-incubation with the antigen.

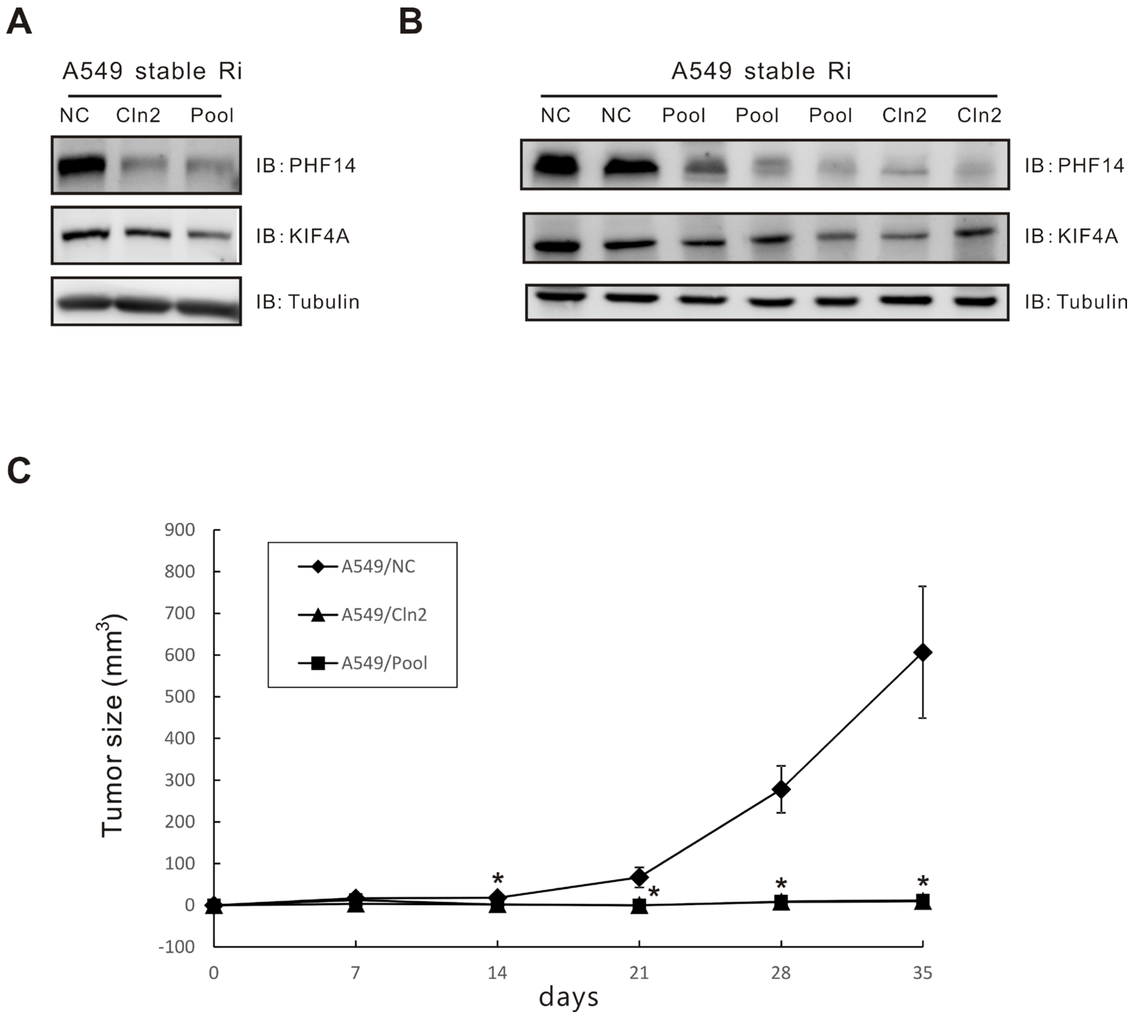

Supplementary Figure 3: PHF14/KIF4A expression and tumor formation in nude mice. A. PHF14/KIF4A expression in A549 control cells (NC) or PHF14 knockdown-cells (A549/Cln2 or PHF14-knockdown pool cells). **B.** PHF14/KIF4A expression in A549 NC, Cln2 or pool cells - formed tumors six weeks after inoculation. **C.** Average tumor size in the mice injected with different cells as indicated. The mean value ± s.d. was calculated from 4-6 samples in each group. * *P*<0.05.

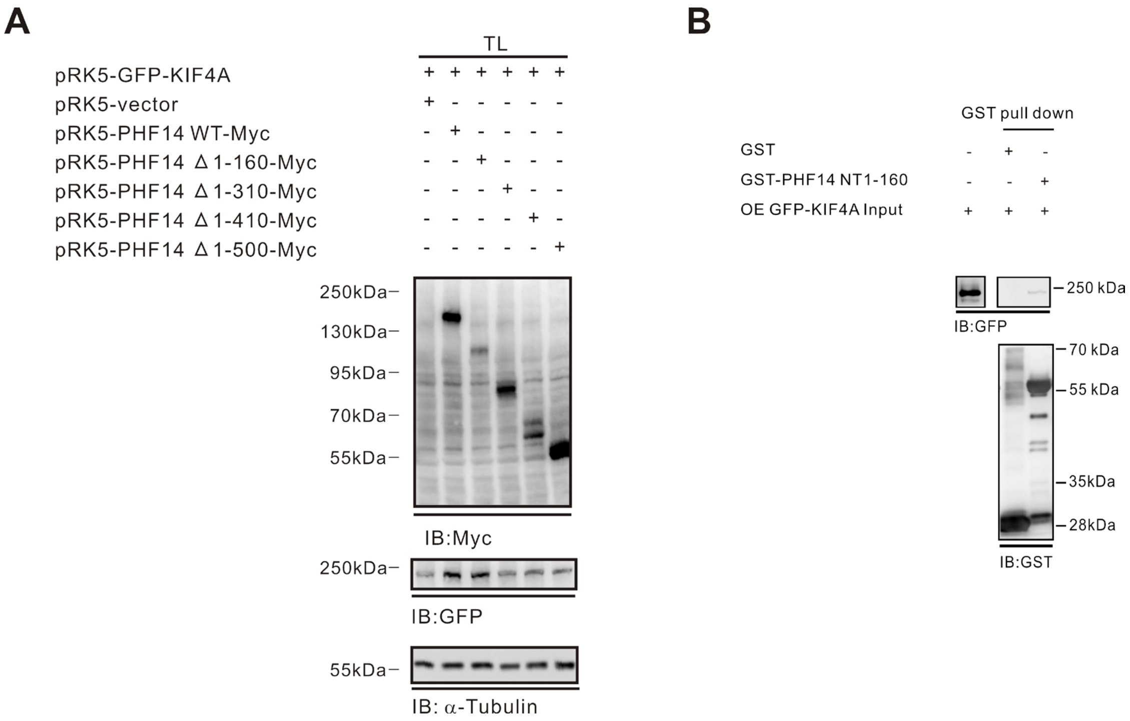

Supplementary Figure 4: PHF14 interacts with KIF4. A. Co-overexpression of myc-tagged PHF14 or its mutants with GFP-tagged WT KIF4A. Different constructs were transfected into the HCT-116 cells. The exogenous PHF14 and KIF4A were detected using an anti-Myc/anti-GFP antibody respectively. α-Tubulin was used as the loading control. **B.** GST pull-down assay. Purified GST-tagged fragments were incubated with 293T cell lysates containing GFP-tagged KIF4A WT.

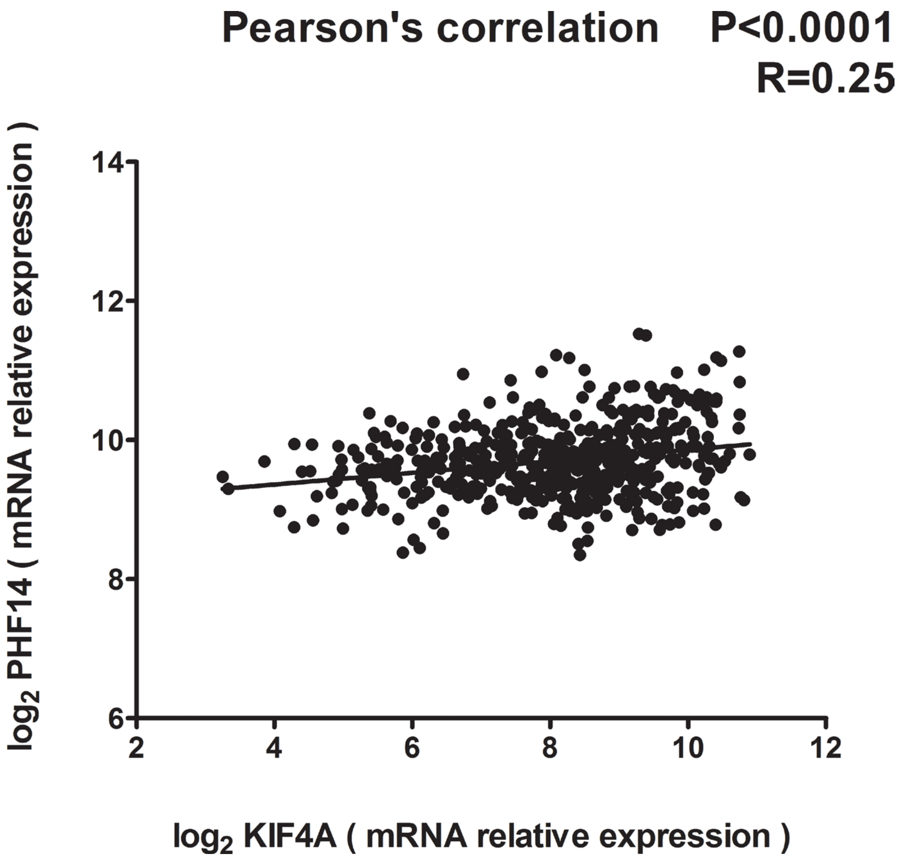

Supplementary Figure 5: PHF14 co-overexpressed with KIF4A. The correlation between PHF14 and KIF4A mRNA expression levels in paired NSCLC samples. RNAseq gene expression profiles were retrieved from TCGA database, n=516, R=0. 246, *P*<0.0001.

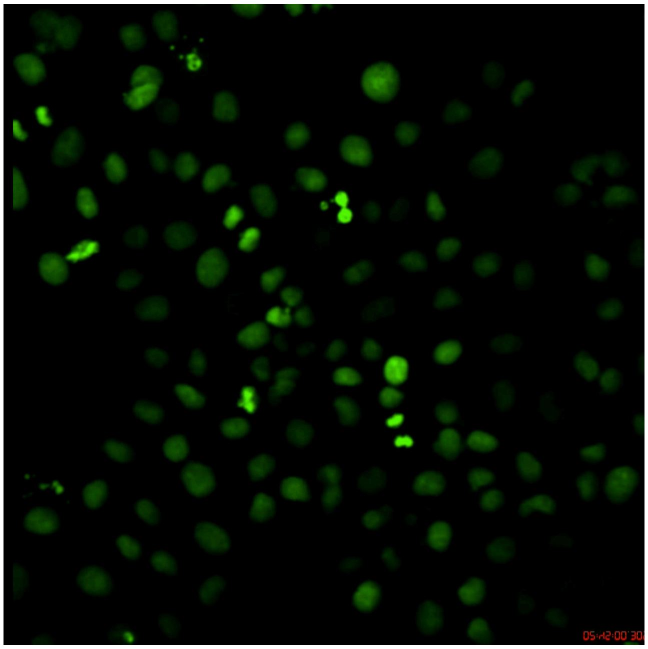

Supplementary Movie 1: Control HeLa cells undergoing mitosis. HeLa cells stably expressing histone H_2B-GFP (green) and transfected with control siRNA. Images were captured every 5 min for about 12h.

See Supplementary Movie 1

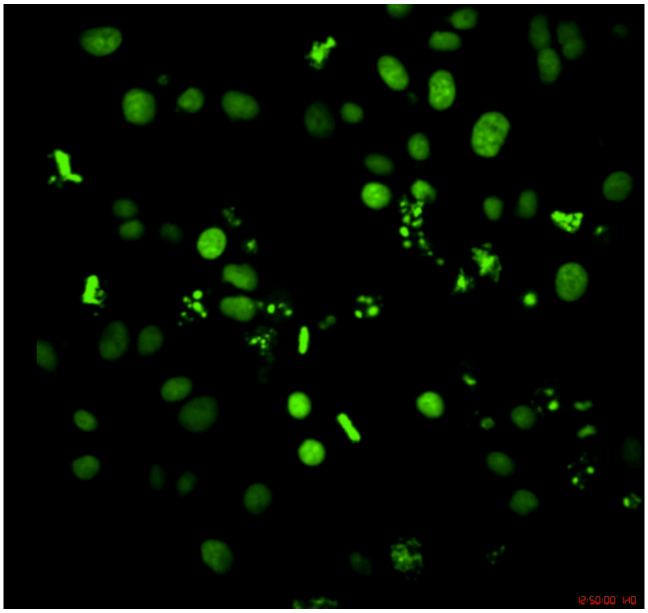

Supplementary Movie 2: Abnormal mitosis in PHF14-knockdown cell. HeLa cells stably expressing histone H_2B-GFP (green) were transfected with siRNA targeting PHF14. Images were captured every 5 min for about 12h.

See Supplementary Movie 2

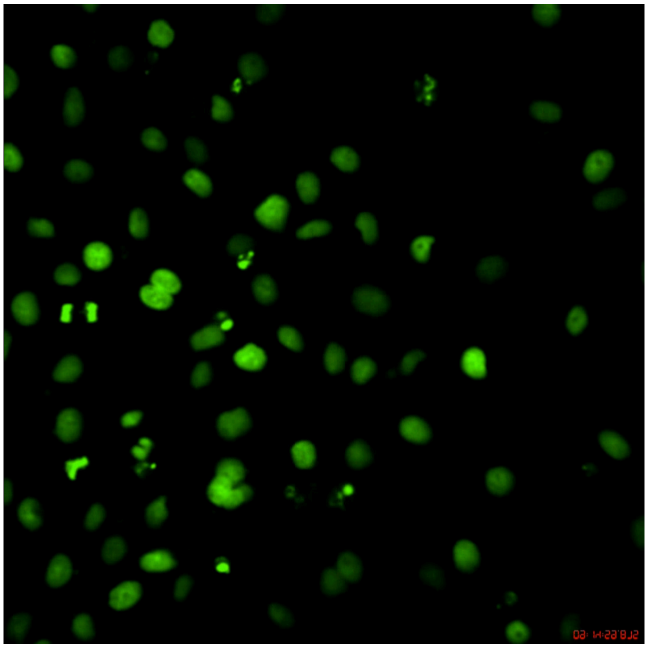

Supplementary Movie 3: Abnormal mitosis in KIF4A-knockdown cell. HeLa cells stably expressing histone H_2B-GFP (green) were transfected with siRNA targeting KIF4A. Images were captured every 5 min for about 12h.

See Supplementary Movie 3

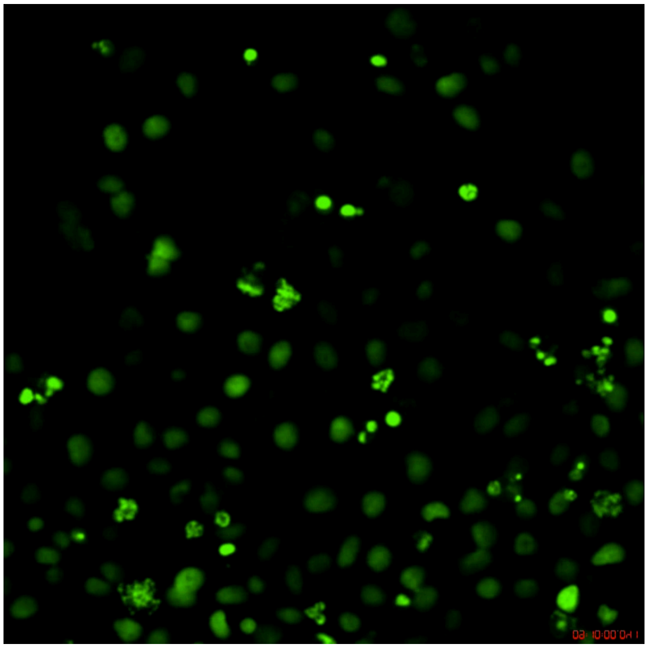

Supplementary Movie 4: Abnormal mitosis in PHF14/KIF4A double-knockdown cell. HeLa cells stably expressing histone H_2B-GFP (green) were transfected with siRNA targeting PHF14 and KIF4A. Images were captured every 5 min for about 12h.

See Supplementary Movie 4

Supplementary Table 1: Mutations of PHF14 detected in lung squamous cell carcinoma and lung adenocarcinoma from TCGA dataset

Disease Type	Participants ID	Gene Name	Sample Name	CDS Mutation	AA Mutation	domain location
Lung Squamous Cell Carcinoma	TCGA-33-4532	PHF14	TCGA-33-4532-01A-01D-1267-08	c.2030G>A	p.S677N	CC2
Lung Squamous Cell Carcinoma	TCGA-34-5239	PHF14	TCGA-34-5239-01A-21D-1817-08	c.2313-6A>T	p.?	PHD3
Lung Adenocarcinoma	TCGA-44-2656	PHF14	TCGA-44-2656-01A-02D-0969-08	c.202G>T	p.E68*	before CC domain1
Lung Adenocarcinoma	TCGA-44-2656	PHF14	TCGA-44-2656-01A-02D-0969-08	c.1437A>T	p.S479S	PHD2
Lung Adenocarcinoma	TCGA-44-2659	PHF14	TCGA-44-2659-01A-01D-0969-08	c.1373G>T	p.C458F	PHD2
Lung Squamous Cell Carcinoma	TCGA-46-3769	PHF14	TCGA-46-3769-01A-01D-0983-08	c.205G>T	p.E69*	before CC domain1
Lung Squamous Cell Carcinoma	TCGA-46-3769	PHF14	TCGA-46-3769-01A-01D-0983-08	c.2004A>G	p.L668L	CC2
Lung Adenocarcinoma	TCGA-49-6742	PHF14	TCGA-49-6742-01A-11D-1855-08	c.1342C>T	p.R448C	PHD2
Lung Adenocarcinoma	TCGA-50-6590	PHF14	TCGA-50-6590-01A-12D-1855-08	c.1318G>T	p.E440*	PHD2
Lung Squamous Cell Carcinoma	TCGA-66-2758	PHF14	TCGA-66-2758-01A-02D-1522-08	c.1520A>G	p.K507R	between PHD2 and CC2
Lung Adenocarcinoma	TCGA-67-3771	PHF14	TCGA-67-3771-01A-01W-0928-08	c.881C>T	p.S294F	between CC1 and PHD1

Supplementary Table 2: Mutations of PHF14 detected in lung adenocarcinoma

Disease Type	Gene Name	Sample Name	CDS Mutation	AA Mutation	domain location
Lung Adenocarcinoma	PHF14	136	c.1718C>T	p.L569F	between PHD2 and CC2
Lung Adenocarcinoma	PHF14	203	c.1818C>T	p.S602F	between PHD2 and CC2
Lung Adenocarcinoma	PHF14	1516	c.1818C>T	p.S602F	between PHD2 and CC2
Lung Adenocarcinoma	PHF14	1574	c.1818C>T	p.S602F	between PHD2 and CC2
Lung Adenocarcinoma	PHF14	1165	c.2797ATG>TGA	p.MK928NE	after PHD4
